# Incidence of cardiac arrests and unexpected deaths in surgical patients before and after implementation of a rapid response system

**DOI:** 10.1186/2110-5820-2-20

**Published:** 2012-06-20

**Authors:** Friede M Simmes, Lisette Schoonhoven, Joke Mintjes, Bernard G Fikkers, Johannes G van der Hoeven

**Affiliations:** 1 Faculty of Health and Social Studies, HAN University of Applied Sciences and the Radboud University Nijmegen Medical Centre, PO Box 6960, 6503, GL, Nijmegen, Netherlands; 2 Scientific Institute for Quality of Healthcare, Radboud University Nijmegen Medical Centre, Nijmegen, Netherlands and Faculty of Health Sciences, University of Southampton, United Kingdom; 3 Department of Intensive Care Medicine, Radboud University Nijmegen Medical Centre, Nijmegen, Netherlands

**Keywords:** Rapid response teams, Outcome and process assessment (health care), General surgery, Hospital mortality, Cardiac arrest

## Abstract

**Background:**

Rapid response systems (RRSs) are considered an important tool for improving patient safety. We studied the effect of an RRS on the incidence of cardiac arrests and unexpected deaths.

**Methods:**

Retrospective before- after study in a university medical centre. We included 1376 surgical patients before (period 1) and 2410 patients after introduction of the RRS (period 2). Outcome measures were corrected for the baseline covariates age, gender and ASA.

**Results:**

The number of patients who experienced a cardiac arrest and/or who died unexpectedly decreased non significantly from 0.50% (7/1376) in period 1 to 0.25% (6/2410) in period 2 *(odds ratio (OR) 0.43, CI 0.14-1.30)*. The individual number of cardiac arrests decreased non-significantly from 0.29% (4/1367) to 0.12% (3/2410) *(OR 0.38, CI 0.09-1.73)* and the number of unexpected deaths decreased non-significantly from 0.36% (5/1376) to 0.17% (4/2410) *(OR 0.42, CI 0.11-1.59)*. In contrast, the number of unplanned ICU admissions increased from 2.47% (34/1376) in period 1 to 4.15% (100/2400) in period 2 *(OR 1.66, CI 1.07-2.55)*. Median APACHE ll score at unplanned ICU admissions was 16 in period 1 versus 16 in period 2 *(NS). Adherence to RRS procedures*. Observed abnormal early warning scores ≤72 h preceding a cardiac arrest, unexpected death or an unplanned ICU admission increased from 65% (24/37 events) in period 1 to 91% (91/101 events) in period 2 *(p < 0.001)*. Related ward physician interventions increased from 38% (9/24 events) to 89% (81/91 events) *(p < 0.001).* In period 2, ward physicians activated the medical emergency team in 65% of the events (59/91), although in 16% (15/91 events) activation was delayed for one or two days. The overall medical emergency team dose was 56/1000 admissions.

**Conclusions:**

Introduction of an RRS resulted in a 50% reduction in cardiac arrest rates and/or unexpected death. However, this decrease was not statistically significant partly due to the low base-line incidence. Moreover, delayed activation due to the two-tiered medical emergency team activation procedure and suboptimal adherence of the ward staff to the RRS procedures may have further abated the positive results.

## Background

Hospitalized patients often show deteriorating vital signs up to 48 h before unexpected death and other serious adverse events [1]. To improve timely recognition and treatment, rapid response systems (RRS) have been introduced. An RRS includes a set of predetermined clinical criteria for assessing patients on a general ward, preferentially at a minimum interval of 12 h [2]. After meeting predefined criteria, a rapid response team has to be activated. This team will evaluate the patient’s physical condition and initiate treatment [3]. RRSs are considered an important tool for improving patient safety and have consequently been implemented and studied worldwide [4,5]. However, great heterogeneity of systems exists concerning the used track and trigger method, the composition of the rapid response team, the rapid response team escalation protocol, and rapid response team interventions. Furthermore, although the usefulness of an RRS appears to be self-evident, research into its effectiveness has yielded equivocal results [6-11]. Despite the presence of an RRS, late rapid response team activation regularly occurs [12-15], suggesting suboptimal adherence of the ward staff with the RRS system. The aim of the current study was to estimate the effects of an RRS, including a two tiered medical emergency team (MET) calling procedure, on the incidence of cardiac arrests and unexpected deaths in surgical patients and to study the adherence of the staff to the RRS procedures.

## Methods

We conducted a retrospective before- after study in surgical patients in a university hospital. The before study was conducted from January 2006 until December 2006 and the after study from April 2007 until April 2009. Patients who were still admitted at the end of the study periods were followed until discharge from the surgical ward. The need for informed consent was waived by the Medical Ethics Committee of district Arnhem-Nijmegen, CMO-nr.: 2005/310.

### Inclusion criteria

We included all patients who stayed in the surgical ward for ≥72 h following general surgery, including central or extensive peripheral vascular surgery, major oncologic surgery, lung surgery, extensive abdominal surgery and trauma.

### RRS implementation

The RRS included the introduction of a medical emergency team (MET) and the use of a single-parameter track and trigger system. The system was based on the following early warning scores (EWS): respiratory rate <8 or >30 per minute, oxygen saturation <90%, systolic blood pressure <90 or >200 mmHg, heart rate <40 or >130 per minute, a decrease of two points in the eye, motor, verbal (EMV) score or if the nurse felt worried about the patient’s condition [16]. The RRS included a 2-tiered MET calling protocol. In the first tier, nurses had to call the ward physician immediately if one of the EWS criteria was met. The ward physician had to evaluate the patient at the bedside within 10 min. In the second tier the ward physicians activated the medical emergency team (MET) immediately if a serious situation existed or if the patient did not stabilize after an initial intervention. The MET was a physician-led team including a critical care physician and a critical care nurse and was accessible 24/7. If the ward physician was unable to visit the patient in time, nurses were expected to activate the MET directly. Ward physicians were junior doctors, present in the hospital 24/7. In case of a cardiac arrest, the cardiac arrest team was called.

During the RRS implementation period, medical and nursing staff were informed about the system. A one-day education program was mandatory for the nursing staff and optional for the medical staff.

Individual pocket-sized, laminated cards displaying the EWS, the SBAR (situation, background, assessment and recommendation) communication protocol and the MET beeper number were handed out to the ward nurses and doctors. Posters with the EWS and the MET beeper number were also displayed in the wards. During the intervention period, newsletters were sent to the medical and nursing staff with feedback on the EWS observation- and ward physician/MET activation rates.

### Measurements

The health status of patients in period 1 and period 2 was compared using the ASA (American Society of Anesthesiologists) classification, a system for assessing the physical status of patients, pre surgery [17].

### Primary outcomes

Primary outcome was the number of patients who experienced a cardiac arrest and/or unexpected death. Unexpected death was defined as death in the surgical ward or death in the ICU after an unplanned ICU admission.

### Secondary outcomes

Secondary outcomes were the number of unplanned ICU admissions, the acute physiological assessment and chronic health evaluation (APACHE II) scores and ICU LOS in patients with an unplanned ICU admission. An unplanned ICU admission was defined as an unexpected ICU admission from the ward, with or without a preceding emergency reoperation. APACHE II scores were estimated within 24 h after unplanned ICU admissions and defined as APACHE II scores at unplanned ICU admission. In addition, we studied the number of deaths with a DNR order.

### Adherence to RRS procedures

Adherence of nurses and doctors was defined as the number of documented abnormal EWS that led to one or more ward physician interventions and to one or more MET interventions. A MET intervention was defined as delayed when at least one abnormal EWS was documented for one or two days preceding the first MET consult. The overall MET dose was defined as the number of MET interventions per 1000 admissions [18].

### Data collection

Data on age, gender, unplanned ICU admissions, APACHE II scores, mortality and unplanned ICU LOS were obtained from the electronic hospital database. Cardiac arrests were retrieved from the cardiac arrest registration database. Subsequently, the recorded EWS, ward physician and MET interventions were collected from the medical records of patients who had a serious adverse event (SAE). An SAE was defined as a cardiac arrest, an unexpected death or an unplanned ICU admission. For this, the medical records of the patients were independently reviewed by two researchers. Although the EWS was not used before implementation of the RRS, documented vital signs and related ward physician interventions were collected according to the EWS criteria. If patients had an emergency reoperation before the unplanned ICU admission, data on EWS preceding the emergency reoperation were collected. Data collection started within 72 h preceding the SAE.

### Statistical methods

Data were analyzed with SPSS, version 17. Comparisons between period 1 and 2 were made using chi-square tests for categorical data, student’s t- test for normally distributed data and the Mann–Whitney *U* test for non-normally distributed data. We also performed a logistic regression analysis in which we adjusted the primary and secondary outcomes for the baseline covariates age, gender and ASA. A *p < 0.05* was considered statistically significant.

## Results

### *Characteristics of the study population*

The two groups differed significantly in age, gender and ASA score (Table 1). In period 1, 2.2% (34/1376) of the patients experienced 43 SAEs and in period 2 3.8% (91/2410) of the patients experienced 107 SAEs. Characteristics of the SAE patients did not differ significantly between the periods (Table 2).

**Table 1:** Characteristics of study population before (period 1) and after (period 2) implementation of an RRS

	**period 1**		**period 2**		**p- value**
	**n = 1376**		**n = 2410**		
age (SD)	55.4	(16.8)	58.0	(16.8)	<0.001*
gender, male (%)	688	(50.0)	1295	(53.7	0.027*
ASA (SD)	2.1	(0.8)	2.2	(0.8)	<0.001*
LOS hospital (IQR)	7.0	(5.0-13.0)	7.0	(5.0-13)	0.265
in-hospital deaths (per 1000 admissions)	18	(13.1)	37	(15.3)	0.573
total ICU admissions (per 1000 admissions)	145	(10.5)	286	(11.9)	0.215
ICU admissions not due to an SAE (%)	111	(8.1)	186	(7.7)	0.701

SD = standard deviation ASA = American Society of Anesthesiologists classification LOS = length of stay in days.

* statistically significant at = <0.05.

**Table 2:** Characteristics of patients with an SAE before (period 1) and after (period 2) implementation of an RRS

	**period 1**		**period 2**		**p- value**
	**n = 30**		**n = 91**		
Age (SD)	61.6	(17.6)	64.7	(12.5)	0.655
Gender, male (%)	21	(70)	65	(71)	0.851
ASA (SD)	2.3	(0.7)	2.5	(0.7)	0.107

SAE = serious adverse event SD = standard deviation ASA = American Society of Anesthesiologists classification.

### Primary outcomes

The percentage of patients who experienced a cardiac arrest and/or who unexpectedly died was 0.50% (7/1376) in period 1, versus 0.25% (6/2410) in period 2 *(odds ratio (OR) 0.43, CI 0.14-1.30)*. The percentage of cardiac arrests was 0.29% (4/1367) versus 0.12% (3/2410)*(OR 0.38, CI 0.09-1.73)* and the number of unexpected deaths was 0.36% (5/1376) versus 0.17% (4/2410)*(OR 0.42, CI 0.11-1.59* (Table 3).

**Table 3:** Cardiac arrests and unexpected deaths before (period 1) and after (period 2) implementation of an RRS

	**period 1**		**period 2**		OR*	**95%CIv for OR**	**p-value**
	**n = 1376**		**n = 2410**				
Patients with cardiac arrests and/or unexpected deaths (%)	7	(0.50)	6	(0.25)	0.43	0.14-1.30	0.134
Number of cardiac arrests (%)	4	(0.29)	3	(0.12)	0.38	0.09-1.73	0.214
Number of unexpected deaths (%)	5	(0.36)	4	(0.17)	0.42	0.11-1.58	0.200

ICU = Intensive care Unit IQR = inter-quartile range LOS = length of stay in days OR = odds ratio * Logistic regressions adjusted for age, gender and ASA 95%CI = 95% confidence interval.

### Secondary outcomes

The percentage of unplanned ICU admissions was 2.47% (34/1376) in period 1, versus 4.15% (100/2410) in period 2 *(OR 1.66, CI 1.07-2.55),* Median APACHE II scores at unplanned ICU admission was 16 in period 1, versus 16 in period 2 (*p = 0.68),* and median ICU LOS was 3.5 days versus 3 days (*p = 0.94)*. The number of deaths with a DNR order was 0.65% (9/1376) versus 0.79% (19/2410) *(OR 1.05 CI 0.46-2.40).*

### Adherence to RRS procedures

37 SAEs were evaluable in period 1, and 101 SAEs in period 2. Observed abnormal EWS within 72 h prior to an SAE increased from 65% (24/37 events) to 91% (91/101 events) (*p < 0.001)*. Ward physician interventions increased from 38% (9/24 events) to 89% (81/91events) (*p < 0.001).* In period 2, ward physicians consulted the MET in 64% of the events (59/91 events), but in 16% (15/91 events) those consultations were seriously delayed for one or two days.

The overall MET dose was 56 per 1000 admissions. The MET was called for 111 patients a total of 134 times. The main trigger that resulted in MET activation was increased respiratory rate and/or decreased oxygen saturation, which was found in 49% (60/122) of the recorded abnormal vital signs. The MET referred the patient to the ICU in 53% (59/134) of the MET reviews. In 20% (12/59 events) the ICU admission followed after MET interventions to stabilize the patient on the ward for one or two days. Of the patients subjected to one or more MET reviews, 9% (10/111 patients) died, of which 1.8% (2/111) unexpected, either on the ICU or on the ward after ICU discharge.

Comparisons between the first and second year of the after study showed no statistical differences in any of the outcomes (data not shown).

## Discussion

We studied the incidence of cardiac arrests and unexpected deaths in surgical patients before and after implementation of an RRS and the adherence of nurses and doctors to the RRS procedures. The number of patients who experienced a cardiac arrest and/or died unexpectedly declined with 50%. Unplanned ICU admissions increased significantly, but the APACHE ll scores and the LOS of those admissions remained almost unchanged. We found a significant improvement in ward physician interventions to almost 90% of the events with an observed abnormal EWS. The MET was consulted in half of the events on the first day when an abnormal EWS was observed.

Although we showed a 50% reduction in the composite end-point cardiac arrest and/or unexpected death, these results were not statistically significant probably due to the low baseline incidence. Reduction of cardiac arrests and unexpected deaths has been shown in studies with a higher baseline incidence compared to our study [19-23]. To show a statistically significant reduction of 50% in the composite end-point cardiac arrests and/or unexpected death, we should have included almost 20.000 patients.

Surprisingly, we found a significant increase of unplanned ICU admissions. Many studies have shown no effect [23-25] whereas others found a decrease in unplanned ICU admissions [19,26]. However, in those studies no information on the adherence to the RRS was provided. The increase of unplanned ICU admissions could be explained because significantly more patients were detected as critically ill and were referred to the ICU. Disappointingly, after implementation of the RRS no significant decrease in the median APACHE II score at unplanned ICU admission or in the median unplanned ICU LOS was found, indicating that ICU referrals apparently were not done at an earlier stage of illness. Our MET dose was relatively high (56 per 1000 admissions) compared to hospitals with a mature RRS (26-56 per 1000 admissions) [18]. However, in our study the MET was not consulted at all or consulted with a delay of one or two days in half of the events. Absent or delayed MET consults may be due to suboptimal adherence of the ward staff to the system. Furthermore, the two-tiered MET calling procedure may have delayed activation. Recent studies have shown that a delayed MET response was independently associated with greater risk of unplanned ICU admissions [15] and hospital mortality [12,13,15]. In addition, we found that in one out of five events, the MET chose to treat the patient on the ward for one or two days, while eventually the patient had to be transferred to the ICU. Therefore, it is also possible that the MET waited too long before transferring these patients to the ICU.

In the medical records of SAE patients, the number of records with reported abnormal vital signs prior to an SAE increased significantly in the after study. A likely explanation is the introduction of the EWS and the training program for nurses. However, EWS recordings were frequently incomplete which is of concern, as monitoring is essential for triage to an appropriate level of care [2]. Adopting an RRS is a complex process that needs time to become established as an integral part of the ward care system [14,27-29]. Even though we found a remarkable improvement in detecting and treating critically ill patients, our results show that further implementation strategies should be developed to improve adherence of the ward nurses and doctors to the RRS procedures and to stimulate the MET to refer the patient to the ICU at an earlier stage of deterioration.

### Strengths and limitations of the study

The outcome ‘unexpected death’ did not take into account patients who died in the operation theatre or patients who died after surgery on the ICU. We also excluded deaths with a DNR order from the primary outcome. Therefore, the outcome ‘unexpected death’ is more informative to evaluate the effects of the RRS compared to the outcome measures ‘in hospital deaths’ or ‘hospital mortality’ used in other studies.

Our study had some limitations to take into consideration. First, in our study a single parameter track and trigger warning system was used. This system is comparable with the MET activation criteria studied by Cretikos et al., which have a positive predictive value of 10% and a sensitivity of 50% [30], implicating that the system would often trigger MET activation while the patient is not at risk for an adverse event. This may have been of influence on the adherence of the ward staff to the system. Second, in the medical records of SAE patients, often no exact time indication was recorded along with observed abnormal EWS. Therefore, timelines were defined in days on which ward physicians and MET were called following an abnormal EWS observation.

Third, we studied the effects of an RRS only in surgical patients as it was expected that those patients would benefit most from the RRS system. However, a recent study showed that an RRS had a greater impact on cardiac arrest and mortality in medical patients compared to surgical patients [31]. Finally, this study was conducted in a single hospital; data may therefore be less applicable to other study populations and settings. However, implementation of an RRS poses challenges in change of behavior, and only progressive accumulation of evidence and experience from different settings and situations will fill the gaps of knowledge in order to adjust the system to the specific needs of a certain setting [14].

## Conclusions

Introduction of an RRS resulted in a non-significant decrease of 50% of patients who experienced a cardiac arrest and/or unexpectedly died. A low base-line incidence and delayed activation due to the two-tiered medical emergency team activation procedure and suboptimal adherence of the ward staff to the RRS procedures may have abated the positive results. Continued education and reinforcement is necessary for an RRS to be successful.

## Abbreviations

APACHE, acute physiological assessment and chronic health evaluation; ASA, American Society of Anesthesiologists; DNR, do not resuscitate; EMV, eye, motor, verbal score; EWS, early warning score; ICU, intensive care unit; LOS, length of stay; MET, medical emergency team; RRS, rapid response system; RRT, rapid response team; SAE, serious adverse event; SPSS, statistical package for the social sciences.

## Competing interests

The authors declare that they have no competing interests.

## Authors’ contributions

FS participated in the design of the study, acquisition, data management and analysis, drafting and preparing the manuscript for publication. LS and BF contributed to the data management and analysis, and in preparing the manuscript for publication. JM and JH contributed to the design of the study, data analysis and preparing the manuscript for publication. All authors read and approved the final manuscript.
